# Pronuclear morphology evaluation in in vitro fertilization (IVF) / intracytoplasmic sperm injection (ICSI) cycles: a retrospective clinical review

**DOI:** 10.1186/1757-2215-6-1

**Published:** 2013-01-03

**Authors:** Alessia Nicoli, Francesco Capodanno, Ilaria Rondini, Barbara Valli, Maria Teresa Villani, Daria Morini, Leonardo De Pascalis, Stefano Palomba, Giovanni Battista La Sala

**Affiliations:** 1 Department of Obstetrics, Gynecology and Pediatrics, Sterility Centre P. Bertocchi, Obstetrics and Gynecology Unit, A.O. S.Maria Nuova, IRCCS, Reggio Emilia and University of Modena, Reggio Emilia, Italy; 2 Department of Psycology, University of Bologna, Bologna, Italy

**Keywords:** Embryo morphology, IVF, ICSI, Pregnancy, Pronuclear morphology, Live-birth

## Abstract

**Background:**

The assessment of the embryo quality is crucial to maintain an high pregnancy rate and to reduce the risk of multiple pregnancy. The evaluation of the pronuclear and nucleolar characteristics of human zygote have been proposed as an indicator of embryo development and chromosomal complement. The aim of the current study was to assess the role of pronuclear morphology evaluation in vitro fertilization (IVF) / intracytoplasmic sperm injection (ICSI) cycles.

**Methods:**

Retrospective clinical analysis on 755 non-elective transfers of only one embryo (ET). Embryo assessment was performed in days 1 and 2. Clinical and biological data were recorded and analyzed according to embryo and/or pronuclear morphology.

**Results:**

Both pronuclear and embryo morphology were significantly related to clinical pregnancy and live-birth rates. No significant difference in clinical pregnancy and live-birth rates was detected when the pronuclear and embryo morphology assessments were combined. Embryo morphology and maternal age were the only independent predictors of favorable outcome by logistic regression analysis.

**Conclusions:**

Pronuclear evaluation is effective to select the best zygotes if ET is performed at day 1, whereas it did not improve the clinical outcomes when combined with embryo morphology evaluation in day 2.

## Background

The selection of the best embryo(s) to transfer is a crucial point in in vitro fertilization (IVF)/ intracytoplasmic sperm injection (ICSI) procedures to maintain a high performance in terms of pregnancy rate and to reduce the risk of multiple pregnancies.

Several methodologies have been proposed and employed for embryo selection, but their clinical efficacy is still extremely debated and considered suboptimal [1-3].

The study of the pronuclear and nucleolar characteristics of human zygote has been proposed as an indicator of embryo development and competence [4-10]. Previous data have suggested that zygote-score could be an efficient tool for embryo selection if combined with embryo morphology evaluation on days 2 and 3 [10,11]. In particular, the morphological characteristics of the human zygote may suggest an increased risk of arrest of the embryo [11]. Moreover, to date, there is not definitive consensus about the clinical efficacy of the zygote pronuclear morphology. In fact, other Authors suggested that embryo morphology has a better prognostic value than zygote-score in embryo selection [12-14], and that pronuclear scoring system has any advantage to evaluate embryo quality [15].

Based on these considerations, the current study was aimed to evaluate if the pronuclear morphology evaluation improves the clinical outcomes in IVF/ICSI when combined with embryo morphology assessment.

## Materials and methods

### Patients

The current was a non-interventional, retrospective single centre review.

Institutional Review Board approval was not required since all couples included underwent a routine IVF/ICSI program in our Reproductive Unit, and no additional/experimental intervention was performed.

All IVF/ICSI cycles performed between April 2008 and November 2010 were reviewed.

All cycles characterized by the transfer of one embryo were selected and included in the final analysis. Were excluded cycles in which with more than one oocyte was fertilized and cleaved.

For any cycle we collected all available clinical and biological data. Specifically, we recorded the patients' characteristics, the controlled ovarian stimulation regimens, the drugs and protocols used for luteal phase support, the characteristics of oocytes retrieved and embryos transfer, the pronuclear and embryo morphology. Implantation, pregnancy, abortion and live-birth rates were also noted.

### Ovarian stimulation and oocyte retrieval

In all cases the controlled ovarian stimulation was achieved using individualized protocols of recombinant-follicle stimulating hormone (rFSH; Gonal F, Serono, Rome, Italy) in down-regulated cycles. A serum estradiol concentrations <50 pg/mL and an absence of follicles having a mean diameter higher than 10 mm were considered as criteria for gonadotropin administration. The ovarian response was monitored by use of serum estradiol assays and serial ultrasonographies.

In presence of at least one follicle with a mean diameter equal or higher than 17 mm was observed, 10,000 IU human chorionic gonadotropin (hCG; Gonasi, IBSA, Milan, Italy) or 250 μg recombinant hCG (Ovitrelle, Serono, Rome, Italy) were intramuscularly administered. Oocyte retrieval was performed 34 to 36 hours after hCG administration by ultrasound-guided transvaginal aspiration.

### Semen preparation

Semen samples were collected by masturbation after 3–5 days of abstinence. The preparation for conventional IVF or ICSI was performed following the World Health Organization standard protocol [16,17].

### Oocyte insemination

For the conventional IVF procedures, oocytes were cultured individually and inseminated in microdrops of fresh medium (Cook IVF, Melbourne, Australia) under mineral oil with 100,000 activated spermatozoa. For ICSI procedure, after the removal of the cumulus and corona cells, oocytes nuclear maturation assessment was performed using an inverted microscope to ensure the injection of metaphase II oocytes exclusively [18].

### Assessment of fertilization/cleavage

Oocyte fertilization was assessed at 18–20 hours (day 1) from insemination/injection and confirmed by the presence of 2 pronuclei (2PN) and the alignment of nucleolar precursor bodies (NPB). At same time, the pronuclear morphological score was assessed [4]. The observation of 2PN was performed using an inverted microscope with Hoffman modulation contrast at ×400 magnification (TE 2000 U, Nikon Corp., Japan). Zygotes with simultaneously juxtaposed and centralized PN, nucleoli of large size and orientated, and orientation of polar bodies in the longitudinal axis of PN were classified as Z1 and considered as the best zygote morphology [4,5,19]. Zygotes having all other configurations were classified as Z2.

ET was performed after 48 hrs (day 2) of embryo culture. During embryo observation five parameters were classified: cleavage symmetry, blastomere shape and size, cytoplasmic aspect, presence of fragmentation and of blastomere multi/micronucleation. Embryos characterized by symmetric cleavage, regular blastomeres sheep and size, absence of any cytoplasmic anomalies such as dark areas, granulations and vacuole, blastomeres multinucleation (≥ 2 nuclei per blastomere) or micronucleation were considered as grade I embryos. Grade II embryos presented slightly irregular blastomeres sheep and size, fragmentation ≤ 10%, and small cytoplasmic vacuoles. Grade III embryos were characterized by asymmetric cleavage, highly irregular blastomeres sheep and size, fragmentation among 10-30%, dark cytoplasm, cytoplasmic vacuoles and multinucleation. Finally, grade IV embryos displayed asymmetric cleavage, severe irregular blastomeres sheep and size, fragmentation among 30-50%, dark cytoplasm with massive presence of vacuoles. ETs of grades I and II embryos were defined as High Quality Embryo ET (HQE ET), whereas ETs of grades III and IV embryos as Poor Quality Embryo ET (PQE ET).

### Embryo transfer and establishment of pregnancy

All patients received intramuscular (100 mg/day; Prontogest; IBSA, Milan, Italy) or transvaginal supplemental progesterone (600 mg/day; Prometrium, Rottapharm, Milan, Italy) for 15 days.

In each case, biochemical pregnancy was determined 12 days after ET by a positive quantitative serum β-hCG assay >10 IU. In case of positive pregnancy test, the hormonal support was continued until 35 days after the ET. Clinical pregnancy was defined as one embryo with heart beat revealed by transvaginal ultrasonography at 5 weeks after ET. Because of only one embryo was transferred in each ET, the clinical pregnancy was equivalent to the embryo implantation.

### Statistical analysis

The normal distribution of continuous variables was evaluated with the use of the Kolmogrov-Smirnov test, and continuous data were expressed as the mean ± standard deviation (SD).

Continuous data were analyzed by Student *t* test for unpaired data. For categorical variables, the Pearson Chi square test was performed; Fisher’s exact test was used for the frequency tables when more than 20% of the expected values were lower than five.

To investigate which of the variables studied could best predict the chances of a clinical pregnancy and live-birth, a binary logistic regression was performed, using embryo quality, insemination technique and pronuclear morphology as predictors.

For all tests, the statistical significance level was set at *P* < 0.05.

Analysis was performed using SPSS version 20 for Microsoft Office.

## Results

In the study period, 2,212 IVF/ICSI cycles were screened and 755 cycles (755/2,212, 34.2%) were included in the final analysis. Specifically, 293 and 462 cycles of IVF (293/755, 38.8%) and ICSI (462/755, 61.2%) programs were studied, respectively. One thousand and two hundred five cycles (1.205/2.212, 54.4%) were excluded because of more then one embryo was transferred, and 252 cycles (252/2.212, 11.4%) because of ET was not performed for fertilization failure or cryopreservation of all oocytes/embryos.

A statistically significant higher clinical pregnancy [17/139 (12.2%) *vs.* 39/616 (6.3%); *P* = 0.017] and live-birth [13/139 (9.4%) *vs*. 30/616 (4.9%); *P* = 0.039] rates were observed in ETs with embryos derived from Z1 zygotes (Z1 ETs) compared to ETs with embryos derived from Z2 zygotes (Z2 ETs). Similarly, a statistically significant higher clinical pregnancy [45/450 (10.0%) *vs.* 11/305 (3.6%); *P* = 0.001] and live-birth [34/450 (7.6%) *vs.* 9/305 (3.0%); *P* = 0.007] rates were observed in HQE ETs compared to PQE ETs (Table 1).

**Table 1:** Clinical outcomes analyzed according to embryo and pronuclear morphologies

	**HQE ET**	**PQE ET**	***P value***
	**(n., %)**	**(n., %)**	
**Total cycles**	450, 59.6	305, 40.4	
**Maternal age (years)**	36.83±3.88	36.48±4.04	0.234
**Clinical pregnancy rate**	44^/450, 9.8	11/305, 3.6	0.001
**Singleton**	42/44, 95.5*	11/11, 100.0	0.471
**Implantation rate**	44/450, 9.8	11/305, 3.6	0.001
**Abortion rate**	9/44, 20.5	2/11, 18.2	0.866
**Live-birth rate**	34/450, 7.6	9/305, 3.0	0.007
**Gestational age at birth (weeks)**	37.8±4.2	39.6±1.3	0.213
**Birth weight (gr)**	2979.3±798.3	3214.6±391.0	0.399
	**Z1 ET**	**Z2 ET**	
	**(n., %)**	**(n., %)**	
**Total cycles**	139	616	
**Maternal age (years)**	36.22±3.71	36.79±3.99	0.123
**Excellent quality embryos**	105/139, 75.5	345/616, 56.0	<0.001
**Clinical pregnancy rate**	16^/139, 12.2	39/616, 6.3	0.017
**Singleton**	16/16, 100.0	37/39, 94.9*	0.356
**Implantation rate**	16/139, 11.5	39/616, 6.3	0.034
**Abortion rate**	2/16, 12.5	9/39, 23.1	0.712
**Live-birth rate**	13/139, 9.4	30/616, 4.9	0.039
**Gestational age at birth (weeks)**	37.3±5.1	38.5±3.1	0.351
**Birth weight (gr)**	2959.6±849.7	3058.4±692.2	0.691

^: one ectopic pregnancy; *: two sets of monozygotic twins.

In Table 2 are showed the clinical pregnancy and live-birth rates per ET according to PN and embryo morphology.

**Table 2:** Clinical pregnancy and live-birth rate analyzed according to pronuclear and embryo morphology

		**Clinical pregnancy rate**			**Live-birth rate**		
		**No**	**Yes**	**Total**	**No**	**Yes**	**Total**
		**(n., %)**	**(n., %)**	**(n., %)**	**(n., %)**	**(n., %)**	**(n., %)**
**HQE ET**	**Z1**	90, 85.7	15, 14.3	105, 100	93, 88.6	12, 11.4	105, 100
	**Z2**	316, 91.6	29, 8.4	345, 100	323, 93.6	22, 6.4	345, 100
**PQE ET**	**Z1**	32, 94.1	2, 5.9	34, 100	33, 97.1	1, 2.9	34, 100
	**Z2**	262, 96.7	9, 3.3	271, 100	263, 97.0	8, 3.0	271, 100
**Total**		700, 92.7	55, 7.4	755, 100	712, 94.3	43, 5.7	755, 100

The distribution pronuclear and embryo morphology was significantly (*P* < 0.05) different between patients who had and who did not had a clinical pregnancy and a live-birth.

The distribution of PN and embryo morphology was significantly different (*P* < 0.05) between patients who had and who did not had a clinical pregnancy and/or a live-birth (Table 2). The HQE-Z1 and PQE-Z2 ETs showed, respectively, the highest and lowest clinical pregnancy and live-birth rates (Table 2). No significant difference between HQE-Z1 and HQE-Z2 ETs and between PQE-Z1 and PQE-Z2 ETs was detected in clinical pregnancy (*P* = 0.093 and *P* = 0.450 for HQE-Z1 *vs.* HQE-Z2 ETs and for PQE-Z1 *vs.* PQE-Z2 ETs, respectively) and live-birth rates (*P* = 0.086 and *P* = 0.997 for HQE-Z1 *vs.* HQE-Z2 ETs and for PQE-Z1 *vs.* PQE-Z2 ETs, respectively) (Table 2).

Finally, using the binary logistic regression to evaluate the best predictor of clinical pregnancy and live-birth only the embryo morphology and the maternal age resulted independent predictors (Table 3).

**Table 3:** Regression analysis using maternal age, embryo quality, insemination technique and pronuclear morphology as predictors of clinical pregnancy and delivery

	***B***	**ES**	***P***	**OR**	**95% CI**	
					**Lower**	**Upper**
**Constant**						
***Clinical pregnancy***	−3.845	0.374	<0.001	0.021		
***Delivery***	−4.084	0.417	<0.001	0.017		
**Maternal age**						
***Clinical pregnancy***	1.263	0.290	<0.001	3.535	2.002	6.244
***Delivery***	1.282	0.326	<0.001	3.604	1.904	6.821
**Insemination technique**						
***Clinical pregnancy***	0.021	0.304	0.946	1.021	0.562	1.854
***Delivery***	0.074	0.341	0.828	1.077	0.552	2.099
**Pronuclear morphology**						
***Clinical pregnancy***	0.481	0.320	0.132	1.618	0.865	3.029
***Delivery***	0.468	0.359	0.192	1.597	0.791	3.227
**Embryo quality**						
***Clinical pregnancy***	1.078	0.353	0.002	2.938	1.470	5.871
***Delivery***	0.974	0.391	0.013	2.648	1.231	5.698

Model for clinical pregnancy prediction: χ^2^(4) = 33.445; *P* < 0.001; R^2^ = 0.106.

Model for delivery prediction: χ^2^(4) = 25.200; *P* < 0.001; R^2^ = 0.093.

## Discussion

To date, the evaluation of pronuclear and embryo morphologies is a common practice in ART laboratories to select the best embryos to transfer. Even if several data regarding the clinical efficacy of pronuclear morphology evaluation have published [4-13,17], the current is the first clinical study reporting reproductive data of patients submitted to the transfer of one embryo because of only one oocyte was fertilized and/or cleaved. In fact, at our knowledge, only Salumets *et al.*[20] included in their analysis reproductive data of a single embryo transferred, even if this was “elective” [20].

The analysis of this specific cohort of patients permitted to exclude *a priori* the bias of embryo selection performed by the operator, even if the overall ART clinical outcomes were, as expected, very poor.

Firstly, our data suggest that both pronuclear and embryo morphology evaluations have a prognostic value in the prediction of clinical pregnancy in IVF/ICSI procedures. Specifically, our data confirm the efficacy of the pronuclear morphology evaluation to predict the clinical pregnancy [4-11,21]. At this regard, current findings highlight that the pronuclear morphology is an important tool not only to individuate abnormal fertilizations (only 1 PN or more than 2 PN) but to select zygotes when ET is performed at day 1, as recently recommended by the European Society of Human Reproduction and Embryology (ESHRE) [1].

Secondly, even if the HQE-Z1 and PQE-Z2 ETs resulted respectively closely related to the highest and lowest reproductive performances, the pronuclear morphology evaluation seemed to give limited additional information for the selection of the embryos to transfer at day 2 [22]. The un-effectiveness of the pronuclear morphology evaluation to improve the embryo selection was observed both in case of high- and low-quality embryos. In fact, the reproductive outcomes did not change after ET of embryos with the same quality deriving from zygotes of different morphology, i.e. HQE-Z1 *vs.* HQE-Z2 and PQE-Z1 *vs.* PQE-Z2.

Finally, our findings demonstrate by binary logistic regression analysis that, after correction for confounders, only the embryo morphology evaluation at day 2 resulted an independent predictors of clinical pregnancy and live-birth.

In conclusion, the current study confirm that pronuclear morphology evaluation is effective to assess the oocyte fertilization and to select the best zygotes if ET is performed at day 1, and suggest that its role for selecting the best embryo to transfer at day 2 is limited. Probably, as suggested by Montag et al. [23], the process of pronuclear formation is a highly dynamic mechanism, hardly resalable in a static evaluation. Further randomized controlled clinical trials on well selected population are needed to confirm or deny our conclusions.

## Competing interests

The authors declare that they have no conflict of interests.

## Authors’ contributions

AN and FC have made substantial contributions to conception and design, BV, DM and MTV have been involved in drafting the manuscript, IR has contributed to the acquisition of the data and revised the manuscript critically, LDP contributed to the analysis and interpretation of data and SP and GB LS have given final approval of the version to be published. All authors read and approved the final manuscript.
